# Selected Neurophysiological, Psychological, and Behavioral Influences on Subjective Sleep Quality in Nurses: A Structure Equation Model

**DOI:** 10.1371/journal.pone.0079529

**Published:** 2013-11-20

**Authors:** Min-Huey Chung, Wen-I Liu, Hui-Ling Lee, Nanly Hsu

**Affiliations:** 1 Graduate Institute of Nursing, College of Nursing, Taipei Medical University, Taipei, Taiwan; 2 School of Nursing, National Taipei University of Nursing and Health Sciences, Taipei, Taiwan; 3 Department of Nursing, Kang-Ning Junior College of Medical Care and Management, Taipei, Taiwan; 4 Nursing Department, Yuanpei University, Hsinchu, Taiwan; University of Medicine & Dentistry of NJ - New Jersey Medical School, United States of America

## Abstract

Few studies have examined relationships among neurophysiological, psychological, and behavioral factors with regard to their effects on sleep quality. We used a structure equation model to investigate behavioral and psychological factors that influence neurophysiological regulation of sleep in shift workers. Using a cross-sectional study design, we tested the model with a sample of 338 female nurses working rotating shifts at an urban regional hospital. The Morningness-Eveningness Questionnaire (MEQ) and short-form Menstrual Distress Questionnaire (MDQ) were used to measure neurophysiological factors involved in morningness-eveningness and menstrual distress. The Sleep Hygiene Awareness and Practice Scale (SHAPS) and Profile of Mood States Short Form (POMS-SF) were completed to measure behavioral factors of sleep hygiene practices and psychological factors of mood states. In addition, the Pittsburgh Sleep Quality Index (PSQI) measured participant's self-reported sleep quality. The results revealed that sleep hygiene practices and mood states mediated the effects of morningness-eveningness and menstrual distress on sleep quality. Our findings provide support for developing interventions to enhance sleep hygiene and maintain positive mood states to reduce the influence of neurophysiological factors on sleep quality among shift workers.

## Introduction

A considerable amount of evidence indicates that the effectiveness of non-drug treatments for insomnia is comparable to that of pharmacologic treatments [1], [2]. Conceptual models for these non-drug treatments comprise behavioral, psychological, and neurophysiological constructs. Behavioral factors related to sleep hygiene may interfere with sleep quality [3]. Sleep hygiene is defined as the establishment of appropriate sleep behaviors that promote sleep quality [4]. These practices include maintaining a stable sleep schedule, not using the bed or bedroom for activities other than sleep, avoiding late-afternoon naps, and avoiding emotionally, physiologically, or cognitively stimulating activities before bedtime [4]. Insomnia patients frequently practice poor sleep hygiene such as smoking or drinking alcohol before bedtime [5]. Sleep hygiene practices may directly affect sleep quality. Moreover, certain people tend to experience numerous changes in their bedtimes, wake-up times, and durations of sleeping periods [6], [7]. Maintaining lifestyle regularity was associated with a decreased risk of depression [8]. Adopting strategies, such as habitual sleep patterns, to improve sleep hygiene may reduce variations in mood. Thus, we hypothesized the following: (H1) a positive association exists between sleep hygiene practices and sleep quality, and (H2) a positive association exists between sleep hygiene practices and mood states.

Psychological factors include reactions to stressful and emotional situations, such as anxiety and mood states, which are significantly associated with excessive worrying or depression during shift work [9]. Psychological symptoms related to neuroticism and depression were found to significantly increase from the late follicular to the late luteal phase in healthy females [10]. Mood states may be affected by menstrual distress. In addition, a previous study showed that poor sleep quality was significantly associated with negative mood states in a large cohort of college students [11]. Thus, we also hypothesized the following: (H3) a positive association exists between mood states and sleep quality, and (H4) a positive association exists between mood states and menstrual distress.

Neurophysiological factors can be considered reactions to circadian variations and menstrual discomfort that are influenced by biological rhythms of sleeping and waking [12], [13]. Morningness and eveningness refer to variations in the circadian phases of a person's endogenous “clock” [14]. These diurnal types can be measured using the Morningness-Eveningness Questionnaire (MEQ), which distinguishes between morning (M)-type and evening (E)-type persons based on preferential behavioral rhythms in their daily activities and habitual sleep patterns [15]. According to the morningness-eveningness conceptual framework, people display different sleep hygiene practices. E-types experience numerous changes in their bedtimes, wake-up times, and durations of sleeping periods [6], [7]. Chung et al. showed that morningness-eveningness is a significant predictor of sleep quality, and suggested that identifying a nurse's diurnal type could assist in correcting poor sleep quality [16]. Furthermore, the morningness-eveningness preference of shift workers significantly influences their mood states [17]. Thus, we proposed the following hypotheses: (H5) a negative association exists between morningness-eveningness and sleep hygiene practices; (H6) a negative association exists between morningness-eveningness and sleep quality; and (H7) a negative association exists between morningness-eveningness and mood states.

Women with severe menstrual symptoms are significantly more likely to use tobacco, drink alcohol heavily, and be overweight than women who do not have menstrual problems [18]. Smoking and drinking heavily close to bedtime are poor sleep hygiene practices [5]. Health behaviors related to sleep hygiene may be related to menstrual distress. In addition, women experiencing menstrual problems reported significantly increased distress, such as insomnia and fatigue [18]. Therefore, we proposed two additional hypotheses: (H8) a positive association exists between menstrual distress and sleep hygiene practice, and (H9) a positive association exists between menstrual distress and sleep quality.

To date, most studies of the contributions of neurophysiological, behavioral, and psychological factors to sleep quality in nurses examined only isolated factors. Consequently, relationships among these factors in nurses remain unclear. In the present study, we used a structure equation model to examine contextual factors underlying sleep quality in nurses. We examined associations among behavioral factors of sleep hygiene, psychological factors of mood states, and neurophysiological factors involved in morningness-eveningness and menstrual distress and their effects on sleep quality.

## Materials and Methods

### Participants

We performed a cross-sectional study using a convenience sample of nurses recruited from an urban regional hospital in Taiwan. The inclusion criteria were as follows: (a) a registered female nurse who provided direct care for patients, (b) a full-time nurse who had worked rotating 8-h shifts for at least 3 months, and (c) no history of psychiatric or neurological disorders. Nurses who were pregnant at any time during the study were excluded. Of the 435 surveys distributed, 359 (82%) were returned, among which 21 were excluded for pregnancy and 2 were censored because of missing data. Thus, 338 completed questionnaires were analyzed. The questionnaire data assessed 19 parameters. Estimations of the response rate and a valid sample size indicated that at least 10∼15 participants were required per measured indicator [19]. Our cross-sectional analysis of the 338 nurses had a statistical power of 0.8.

### Ethical Considerations

Our study was approved by the Institutional Review Board at Taipei Medical University Hospital. The purpose, content, duration, and methods of the study, as well as the guidelines for protecting their fundamental rights, were explained to potential participants before the commencement of the study during a ward meeting. All participants provided written consent. Data were anonymously collected, remained confidential, and were used for academic research purposes only. If a participant chose to withdraw during the course of the study, the researcher fully respected her right to do so, and guaranteed that her interests would not be harmed as a result.

### Instruments

#### Morningness-Eveningness Questionnaire (MEQ)

The MEQ is the most widely used questionnaire in chronopsychological research. It measures self-reported sleeping and waking habits [20]. People who display a preference for waking up early in the day are referred to as M-types. Conversely, E-types prefer sleeping later, and experience better functioning in the evening [21]. The MEQ contains 19 items, with possible total scores ranging 16∼86. Our participants' diurnal preferences were determined based on their MEQ scores. People who scored ≥59 points were categorized as M-types, and those scoring ≤41 points were categorized as E-types. Respondents scoring 42∼58 points were considered neither type [20]. An acceptable internal consistency of 0.71 was reported for the Chinese version of the MEQ [22]. Cronbach's α for our study was acceptable at 0.74, with a split-half reliability of 0.70.

#### Short-Form Menstrual Distress Questionnaire (MDQ; MDQ-SF)

The MDQ assesses a woman's feelings during her menstrual cycle [23]. The questionnaire assesses 8 symptoms covered by 47 items. The MDQ demonstrated acceptable reliability and validity in previous studies, and the average α coefficient for each subscale [24] ranged 0.64∼0.88. The MDQ-SF used in our study contains 22 items that assess 4 symptoms, including pain, water retention, autonomic reaction, and negative effects, on a 5-point scale [25]. A higher score indicates greater discomfort during menstruation. In our study, the MDQ-SF had a Cronbach's α of 0.92 and a split-half reliability of 0.93.

#### Sleep Hygiene Awareness and Practice Scale (SHAPS)

The SHAPS compares sleep hygiene practices among insomnia patients with those of good sleepers [26]. The sleep hygiene practice section contains 19 self-reported items that inquire about how many days per week a participant practices certain activities, including napping, regular use of sleep medications, waking up at the same time every day, and smoking or drinking alcohol in the evening. Responses are scored on a 7-point Likert scale ranging from 0 (very beneficial) to 7 (very disruptive). Possible total scores range 0∼133, and a higher score indicates poorer sleep hygiene practices [26]. Acceptable test-retest reliability was reported for the SHAPS [27]. In our study, the Chinese version of the SHAPS had a Cronbach's α of 0.71 and a split-half reliability of 0.74 after the “sleep disturbance through temperature” item was discarded.

#### Short-Form Profile of Mood States (POM-SF)

We used the POMS-SF to assess mood states of participants during the week prior to data collection [28]. The 30-item questionnaire consists of tension, depression, anger, fatigue, confusion, and vigor subscales. Each subscale contains 5 items, each of which is scored on a 5-point Likert scale ranging from 0 (not at all) to 4 (extremely). Possible global scores range 0∼120, and a higher score represents a higher level of mood disturbance [28], [29]. The internal consistency of the English version of the POMS-SF [28] was shown to be at least 0.76. The Chinese version of the POMS-SF respectively had internal consistency indices of 0.75∼0.99 and 0.71∼0.91 for patients with and those without pain. The content validity index was 0.92 [30]. Cronbach's α for the 6 POMS-SF subscales in our study ranged 0.88∼0.90.

#### Pittsburgh Sleep Quality Index (PSQI)

The PSQI measures self-reported sleep quality and sleep disturbances during the preceding month [31]. Its 7 components assess subjective sleep quality, sleep latency, sleep duration, sleep efficiency, sleep disturbances, use of sleeping medications, and daytime dysfunction. Each component is scored from 0 to 3, yielding a global score ranging 0∼21, with a higher score indicating poorer sleep quality [31]. The reliability and validity of the PSQI were demonstrated in primary insomnia patients of various populations [32]. The PSQI has good internal consistency, with Cronbach's α ranging 0.77∼0.83 [33]. In studies of shift workers, Cronbach's α for the PSQI was 0.70, indicating acceptable reliability [34]. The Chinese version of the PSQI demonstrated an internal consistency of 0.76 [35]. In our study, the global PSQI had a Cronbach's α of 0.71, indicating acceptable reliability.

### Statistical Analysis

**Figure 1:**
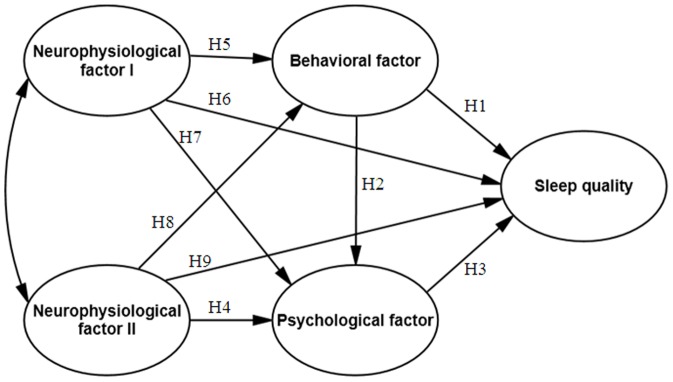
Schematic representation of relationships among exogenous and endogenous variables (solid arrows represent hypothesized paths) that were examined using the structure equation model.

All analyses were performed using the SPSS computer software, vers. 20.0 for Windows (SPSS, Chicago, IL). Results of comparisons with a *p* value of <0.05 were considered to represent statistically significant differences. Scores for the SHAPS, POMS-SF, MEQ, MDQ, and PSQI were compared using Student's *t*-test, and their relationships were explored using a Pearson correlation analysis. Fitness indices of the SPSS AMOS 20.0 (IBM, Armonk, NY) were used to estimate how well the hypothesized model fit the actual data. A model that adequately fits the data can provide a plausible representation of the causal structure of the study. Path diagrams were used to graphically represent the underlying structure of the variable effects in the hypothetical model (Figure 1). We used latent variable modeling with item parceling to determine significant predictors of sleep quality. The technique of parceling with an average of continuous multi-item scales was used to construct item parcels. For model fit and parameter estimations, a maximum-likelihood (ML) estimation was used, and measurement errors were included in the model. Measurement errors were computed for each latent variable as (1−α)×δ^2^, where α is the reliability estimate (Cronbach's α coefficient) of the variable, and δ^2^ is the variance of the mean score of the latent construct as previously described [36]. Parceled data are a few parameters to be estimated in defining a construct model and are considered to less likely have correlated residuals or multiple cross-loadings [37]. Therefore, parceled data may result in reductions in various sources of sampling error.

To determine the ML estimation of the model, values for the goodness-of-fit index (GFI), the adjusted GFI (AGFI), the normed-fit index (NFI), the comparative fit index (CFI), and the root mean squared error of the approximation (RMSEA) were calculated. The GFI, AGFI, NFI, and CFI should achieve values of ≥0.90, and the RMSEA should be at least 0.08 if the model fits the data well [38]. We tested the hypothesis that psychological and behavioral factors predicted sleep quality through interactions with neurophysiological factors using a previously described method [39]. The Sobel test was used to estimate the significance of mediation effects [40]. In addition, the mediation effect on the product-of-coefficients model was assessed with asymmetric confidence intervals using the PRODCLIN2 program [41]. The 95% confidence intervals (CIs) did not include 0 to indicate the existence of mediation effects.

## Results

Our study included 338 female shift nurses aged 27.85±5.15 years, with an average of 3.52±4.29 years of professional nursing experience. The average body-mass index was 20.90±3.20 kg/m^2^. Most participants were unmarried (82.0%). The majority of participants regularly drank tea or coffee (78.1% and 61.5%, respectively). Most of the participants were categorized as neither circadian type (71.9%). In Table 1, significant differences were found in sleep quality between groups of shift schedule, menstrual distress, sleep hygiene practice, and mood states. Therefore, the PSQI score in the structure equation model was measured by 3 shift groups. Table 2 shows that significant positive correlations existed for scores of sleep quality with mood states, sleep hygiene, and menstrual distress. However, a significant negative correlation was observed for scores of sleep quality with morningness-eveningness.

**Table 1:** Demographic characteristics, circadian types, menstrual distress, sleep hygiene, mood states, and sleep quality (*N* = 338).

Variable	Sleep quality (mean ± SD)	*p* value
Age (years)		
≥26	8.09±3.04	0.206
<26	7.63±3.45	
Nursing experience (years)		
≥1.79	8.05±3.02	0.220
<1.79	7.61±3.52	
Body-mass index (kg/m^2^)		
≥24	7.92±3.28	0.238
<24	7.31±3.32	
Marital status		
Single	7.92±3.30	0.284
Married	7.42±3.23	
Consumption of tea		
Yes	7.86±3.36	0.726
No	7.71±3.01	
Consumption of coffee		
Yes	7.80±3.26	0.865
No	7.87±3.34	
Shift schedule		
Day shift	7.39±3.21	<0.05
Evening shift	7.82±3.62	
Night shift	8.69±2.94*	
Circadian type		
Morning	6.53±2.63	0.064
Neither	7.83±3.24	
Evening	8.30±3.57	
Menstrual distress score		
≥17	8.80±3.39	<0.001
<17	6.80±2.83	
Sleep Hygiene Practice score		
≥31	9.12±3.19	<0.001
<31	6.41±2.77	
Profile of Mood States score		
≥46	8.97±3.20	<0.001
<46	6.40±2.87	

The dichotomized group was based on the median of each independent variable except for the body-mass index; SD, standard deviation of the mean;

* *p*<0.05 vs day shift.

**Table 2:** Correlations among selected neurophysiological, psychological, and behavioral factors, and sleep quality (*N* = 338).

	BF	PF	NF1	NF2	SQ
BF (Sleep Hygiene Practice score)	1.00				
PF (Mood States score)	0.32*	1.00			
NF1 (morningness-eveningness score)	−0.29**	−0.15**	1.00		
NF2 (menstrual distress score)	0.28*	0.41*	−0.13*	1.00	
SQ (sleep quality score)	0.49**	0.43*	−0.13*	0.36*	1.00

BF, behavioral factors (sleep hygiene practice); PF, psychological factors (mood states); NF1, neurophysiological factor I (morningness-eveningness); NF2, neurophysiological factor II (menstrual distress); SQ, sleep quality.

* *p*<0.05;

** *p*<0.001.

**Figure 2:**
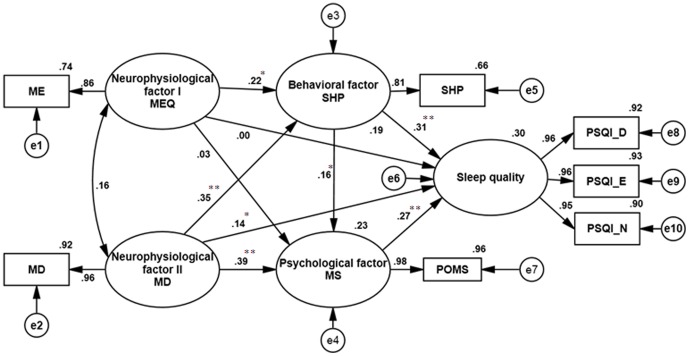
Model of selected behavioral, psychological, and neurophysiological factors that affect sleep quality. Model fit indices: χ^2^/d.f. = 2.335, goodness of fit index (GFI) = 0.981, adjusted GFI (AGFI) = 0.947, comparative fit index (CFI) = 0.991, normed-fit index (NFI) = 0.985, root mean squared error of the approximation (RMSEA) = 0.063. Abbreviations: SHP, Sleep Hygiene Practices; POMS, Profile of Mood States; ME, morningness-eveningness; MD, menstrual distress; PSQI, Pittsburgh Sleep Quality Index; PSQI_D, PSQI in the day shift; PSQI_E, PSQI in the evening shift; PSQI_N, PSQI in night shift; error (e). * *p*<0.05. ** *p*<0.001.

As shown in Figure 2, the statistical goodness of fit of the structural model, denoted by the *χ^2^* value, was 2.335 (*d.f.* = 10, *p* = 0.010). The GFI was 0.981, the AGFI was 0.947, the NFI was 0.985, the CFI was 0.991, and the RMSEA was 0.063. In the model, sleep hygiene practice (β = 0.31, *p*<0.001), menstrual distress (β = 0.14, *p*<0.05), and mood states (β = 0.27, *p*<0.001) significantly predicted sleep quality. However, sleep quality was not significantly affected by the variable of morningness-eveningness (β = 0.00, *p* = 0.919). Table 3 summarizes direct, indirect, and total effects of dominant factors of sleep quality. According to path coefficients, sleep hygiene practices exhibited the strongest direct effects on sleep quality. Menstrual distress, despite showing a weaker direct effect than sleep hygiene, exhibited the strongest total effect on sleep quality.

**Table 3:** Direct, indirect, and total effects of dominants on sleep quality.

Variable	Total effect			Direct effect			Indirect effect		
	BF	PF	SQ	BF	PF	SQ	BF	PF	SQ
NF1 (morningness-eveningness)	0.217	0.062	0.083	0.217	0.028	−0.001	-	0.034	0.084
NF2 (menstrual distress)	0.345	0.441	0.364	0.345	0.386	0.139	-	0.055	0.225
BF (Sleep Hygiene Practice)	-	0.158	0.353	-	0.158	0.311	-	-	0.042
PF (Mood States)	-	-	0.267	-	-	0.267	-	-	-

BF, behavioral factors (sleep hygiene practice); PF, psychological factors (mood states); NF1, neurophysiological factor I (morningness-eveningness); NF2, neurophysiological factor II (menstrual distress); SQ, sleep quality.

In Figure 2, all path coefficients were significant and supported all of the hypotheses, except for H6 and H7. The 4 equations of indirect effects were used to examine mediation effects. As shown in Table 4, the NF1→BF→SQ, NF2→BF→SQ, and NF2→PF→SQ equations were used to estimate indirect effects of NF on sleep quality. Mediating effects exerted through these paths and the BF→PF→SQ equation were significant (Sobel test, *p*<0.05), and the 95% CI did not include 0, indicating mediation effects of behavioral and psychological factors on sleep quality.

**Table 4:** Results of the mediation effect analysis.

Equation	Relationship	Regression weight	Standard error	Sobel test	95% Asymmetrical coefficient interval
NF1→BF→SQ	NF1→BF	0.224	0.078	2.40*	(0.06, 0.44)
	BF→SQ	1.005	0.230		
NF2→BF→SQ	NF2→BF	0.345	0.068	3.31*	(0.15, 0.62)
	BF→SQ	1.005	0.230		
NF2→PF→SQ	NF2→PF	0.386	0.059	3.79*	(0.15, 0.55)
	PF→SQ	0.861	0.185		
BF→PF→SQ	BF→PF	0.158	0.072	1.98*	(0.01, 0.31)
	PF→SQ	0.861	0.185		

BF, behavioral factors (sleep hygiene practice); PF, psychological factors (mood states); NF1, neurophysiological factor I (morningness-eveningness); NF2, neurophysiological factor II (menstrual distress); SQ, sleep quality.

* *p*<0.05.

## Discussion

We tested a comprehensive conceptual framework for predicting the sleep quality of nurses. Statistical results supported the hypothesis that neurophysiological factors influence sleep by mediating psychological or behavioral regulation of normal sleep. Because there is limited empirical data from previous studies supporting the applicability of such a conceptual model, our results provide evidence of the applicability of the conceptual model among nurses. This is the first study to use a conceptual model to investigate behavioral, psychological, and neurophysiological factors underlying sleep quality in nurses. Our findings warrant further study of the conceptual model in other types of healthcare providers, in different clinical settings, and in different cultures.

Our findings demonstrated that behavioral, psychological, and neurophysiological factors are significant predictors of sleep quality. In Table 3, our results showed that behavioral factors, such as sleep hygiene practices, caused direct and indirect effects on sleep quality. Moreover, behavioral factors appeared to be the strongest predictors of sleep quality (β = 0.31, *p*<0.001) as shown in Figure 2. Behavioral factors may be important predictors of sleep quality in nurses. Although it contrasts with those of some previous studies [12], [42], this finding is consistent with those of others [43], [44]. Sleep hygiene indicates that one maintains a regular sleep/wake pattern and controls all environmental factors that may interfere with sleep [4]. Nurses may know that shift patterns induce further irregular sleep-wake times; they usually try to practice good sleep hygiene. If they have difficulty sleeping at night because of work, they might limit themselves to more hours in bed at other times to reduce sleepiness on the night shift. The misalignment of biological rhythms with social rhythms imposed by one's work schedule may explain why neurophysiological factors influence sleep quality through mediation of the regulation of sleep hygiene practice.

Table 2 shows that mood states were positively correlated with menstrual distress and the PSQI score, such that poorer mood states were associated with higher menstrual distress and poorer sleep quality. Mood states also exerted direct influences on sleep quality. These results are consistent with those of a previous study, which showed that negative moods, such as depression and anxiety, were associated with nighttime sleep [45]. Furthermore, mood states mediated the effects of sleep hygiene practices on sleep quality as shown in Figure 2 and Table 4. Mood states may be affected by environmental and behavioral factors that are considered part of sleep hygiene practices, such as lighting, noise, temperature, hunger, caffeine intake, and the use of alcohol and tobacco. However, determining whether related factors of mood states are associated with sleep hygiene in predicting sleep quality requires further study.

Menstrual distress, sleep hygiene, and mood states exerted direct effects on sleep quality as shown in Table 3. The regulation of sleep quality may be more affected by menstrual distress (total effect = 0.372) than by morningness-eveningness (total effect = 0.067; Table 3). Nurses likely experience poor sleep quality through indirect effects of neurophysiological factors, especially menstrual distress (β = 0.35, *p*<0.001; Figure 2). These findings are consistent with those of previous studies in which higher scores for menstrual distress correlated with higher PSQI scores, representing poorer sleep quality [46]. In addition, our results also showed that menstrual distress affected sleep quality by mediating mood states, and menstrual distress had stronger total effects on sleep quality. Menstrual distress indicates the intensity of symptoms that female nurses experience. Our results support a previous study [47] which showed that women with menstrual distress were significantly more likely to report frequent depression and insomnia than those without menstrual-related problems. These results imply that the effects of interventions on menstrual distress are important factors among female shift workers.

The morningness-eveningness characteristics of nurses were associated with their mood states as shown in Table 2. This result is consistent with mood changes in morningness-eveningness chronotypes in healthy individuals [48]. However, our results showed that mediation effects of mood states were not significant between morningness-eveningness and sleep quality. One possible reason is that the neurophysiological factor of menstrual distress and behavioral factors of sleep hygiene were added to the structure equation model to decrease mediation effects. In Table 2, negative correlations were also found between mornings-eveningness and sleep quality. In regard to morningness-eveningness, participants who belonged to the E-type had worse sleep quality. This result is consistent with a previous study [49], which indicated that eveningness was associated with a shorter time in bed during the week. However, our results showed that morningness-eveningness had diminished effects on sleep quality through the mediator of sleep hygiene practice (β = 0.001, *p* = 0.919) in Figure 2. The stronger effects of sleep hygiene practice on sleep quality (β = 0.31, *p*<0.001) may explain why our findings were not consistent with those of a study by Chung et al. [16]. Clarification of the causal relationship between diurnal type and menstrual distress warrants further investigation.

Certain limitations to our findings should be considered. Any causality suggested by the path analysis must be interpreted cautiously because of the cross-sectional design of our study. The POMS assesses mood states during the preceding week, whereas the PSQI assesses sleep quality during the preceding month. Thus, this difference in time frames may have caused some bias. The subjective scale of the SHAPS was not designed for shift workers, and does not address behaviors for promoting sleep that may be unique to shift workers, such as limited exposure to light during the night shift. In addition, not all neurophysiological and psychological factors were evaluated in our study. In future studies, researchers should consider differences in bedtimes resulting from habits or behaviors specifically related to the participants' working conditions. Night shift workers may drink relatively high amounts of caffeine to assist them in staying awake during the night. Therefore, the amount and timing of caffeine ingestion should be recorded in future studies of shift workers. Our sample consisted of young nurses with relatively little work experience, all drawn from a single medical facility. These features of our study group limit the generalization of the results regarding the effects of diurnal preference, menstrual distress, mood states, and sleep hygiene on sleep quality in nurses working rotating shifts.

## Conclusions

We examined possible pathways of associations among behavioral, psychological, and neurophysiological factors that mediate sleep quality in nurses. We evaluated the fitness of a conceptual model to clarify factors that influence sleep quality in nurses. Our results support the hypothesis that neurophysiological factors, such as menstrual distress and morningness-eveningness, influence sleep quality in nurses by mediating behavioral (sleep hygiene) and psychological factors (mood states). Future longitudinal studies are warranted to explore possible causal links between these factors. The clinical significance of our findings is that the effects of menstrual distress and morningness-eveningness on sleep quality may be reduced by practicing good sleep hygiene and maintaining positive mood states.
